# 

**Published:** 1894-04

**Authors:** 


### CONTENTS.

ORIGINAL COMMUNICATIONS

Vaginal Hysterectomy for Malignant Disease of the Cervix, and Uterine Myoma. By L. S. McMurtry, M. D., Louisville, Ky.... 65

Two Recent Cases of Excision of the Vermiform Appendix for Chronic Relapsing Appendicitis in the Interval. By Thomas G. Morton, M. D., Philadelphia.. 71

Treatment of Gunshot Wounds. By Willis F. Westmoreland, M. D., Atlanta, Ga.. 74

SOCIETY REPORTS—

Philadelphia Academy of Surgery.... 80

CORRESPONDENCE—

New York Letter.... 87

LEGAL REQUIREMENTS—

For the Practice of Medicine in the United States.... 94

EDITORIAL—

The Medical Association.... 99

Small-Pox in Atlanta.... 99

College Commencements.... 101

The First American Symphyseotomy.... 103

SELECTIONS AND ABSTRACTS—

Surgery.... 104

Skin Diseases and Syphilis.... 109

Practical Chemistry and Pharmacy.... 111

General Medicine and Therapeutics.... 115

MEDICAL ITEMS.... 118

BOOK REVIEWS.... 120

MEETINGS.... 123

ADVERTISERS' INDEX TO ADVERTISEMENTS.... xii

CLASSIFIED INDEX TO ADVERTISEMENTS.... xxxv

ITEMS OF INTEREST.... x, xi

JOURNAL FOR 1894.... vii

PREMIUM OFFERS.... xxxiv

